# Three-day dendritic cells for vaccine development: Antigen uptake, processing and presentation

**DOI:** 10.1186/1479-5876-8-90

**Published:** 2010-09-28

**Authors:** Maja Bürdek, Stefani Spranger, Susanne Wilde, Bernhard Frankenberger, Dolores J Schendel, Christiane Geiger

**Affiliations:** 1 Helmholtz Zentrum München, German Research Center for Environmental Health, Institute of Molecular Immunology, Marchioninistr. 25, 81377 München, Germany

## Abstract

**Background:**

Antigen-loaded dendritic cells (DC) are capable of priming naïve T cells and therefore represent an attractive adjuvant for vaccine development in anti-tumor immunotherapy. Numerous protocols have been described to date using different maturation cocktails and time periods for the induction of mature DC (mDC) *in vitro*. For clinical application, the use of mDC that can be generated in only three days saves on the costs of cytokines needed for large scale vaccine cell production and provides a method to produce cells within a standard work-week schedule in a GMP facility.

**Methods:**

In this study, we addressed the properties of antigen uptake, processing and presentation by monocyte-derived DC prepared in three days (3d mDC) compared with conventional DC prepared in seven days (7d mDC), which represent the most common form of DC used for vaccines to date.

**Results:**

Although they showed a reduced capacity for spontaneous antigen uptake, 3d mDC displayed higher capacity for stimulation of T cells after loading with an extended synthetic peptide that requires processing for MHC binding, indicating they were more efficient at antigen processing than 7d DC. We found, however, that 3d DC were less efficient at expressing protein after introduction of *in vitro *transcribed (*ivt*)RNA by electroporation, based on published procedures. This deficit was overcome by altering electroporation parameters, which led to improved protein expression and capacity for T cell stimulation using low amounts of *ivt*RNA.

**Conclusions:**

This new procedure allows 3d mDC to replace 7d mDC for use in DC-based vaccines that utilize long peptides, proteins or *ivt*RNA as sources of specific antigen.

## Background

The benefit of dendritic cells (DC) as adjuvants to induce tumor-specific cytotoxic T cells as well as helper T cells has been demonstrated in animal experiments and initial human trials [1,2]. In different tumor vaccines that were successfully applied in mice, mature DC (mDC) were used that were loaded with tumor antigens, supplied in various forms, including tumor extracts, short peptides or antigen-encoding RNA [3,4]. Several clinical trials using DC as tumor-vaccines have also been performed, where an increased T cell response against tumor-associated antigens could be observed [5].

DC are the most potent antigen-presenting cells for the stimulation of naïve T cells [6]. Immature DC (iDC) patrol peripheral tissues and take up antigens via macropinocytosis, phagocytosis or receptor-mediated endocytosis. After uptake of antigen, iDC process and present antigen-derived peptides on their MHC molecules. Since DC have the ability for cross-presentation, exogenous antigens can be presented on MHC-II as well as on MHC-I molecules [7]. Presentation of antigens by iDC leads to T cell anergy, deletion of T cells or the induction of IL-10-secreting T regulatory cells [8,9]. Following antigen uptake, iDC convert to a mature phenotype, characterized by the upregulation of different cell surface molecules, such as CD40, CD80 and CD83 [10]. These mDC also show higher expression of the chemokine-receptor CCR7, which plays an important role for DC homing to lymph nodes [11]. Upon arrival in the lymph nodes, antigen-loaded mDC are able to prime naïve T cells, which then exit the lymph nodes after antigen-encounter. The primed effector T cells can recognize and eliminate specific target cells in the periphery.

Different protocols for the generation of DC have been described to date. *In vitro*, DC can be developed from CD34^+ ^precursor cells or CD14^+ ^monocytes [10,12]. Monocytes can be enriched from peripheral blood mononuclear cells (PBMC) via plate adherence, by the use of anti-CD14 antibodies or by elutriation of leukapheresis products. iDC are usually induced by stimulation with GM-CSF and IL-4 [13,14]. It has also been shown that IL-4 could be replaced by IL-15, leading to the differentiation of monocytes into cells with properties of Langerhans cells [15-17]. Furthermore, DC can also be induced in the presence of IFN-β and IL-3 [18,19]. The induction of mDC can be initiated by several different stimuli, including microbial components (e.g. LPS as a Toll-like receptor 4 ligand), proinflammatory cytokines, viral-like stimuli [e.g. poly (I:C)] or T cell-derived molecules (e.g. CD40L) [16,18,20-24]. Depending on the composition of the maturation cocktails, mDC show different stimulatory and polarizing capacities on naïve T cells.

Most protocols for the generation of mDC require approximately one week of cell culture. As such, Jonuleit and colleagues induced mDC on day five to six of a seven-day culture period by adding a four-component maturation cocktail (hereafter 4C cocktail), containing TNF-α, IL-1β, IL-6 and PGE_2 _[22], that is commonly used for the induction of DC maturation. It has been shown that mDC could also be generated within two days [25,26]. These "fast DC" were generally able to prime naïve T cells or stimulate effector cells [25,27,28]. The faster development of mDC may better reflect the situation *in vivo *[29].

In this study, we performed a systematic comparison of 3d and 7d mDC in terms of phenotype, chemokine-directed migration, antigen uptake and subsequent stimulation of cytotoxic T lymphocytes (CTL) after incubation with exogenous peptides or loading with antigen via electroporation. Because different forms of antigen are considered for use in DC-based vaccine development, it was important to demonstrate that mDC prepared in a three-day protocol would have antigen processing capacity comparable to the well known properties of 7d mDC.

## Materials and methods

### Peptides, antibodies and reagents

The short MART-1/Melan-A_26-35 _peptide (ELAGIGILTV) (purchased from Metabion, Martinsried, Germany) and the long MART-1/Melan-A peptide (GSGHWDFAWPWGSGLAGIGILTV) (purchased from Biosyntan, Berlin, Germany) were reconstituted in 50% DMSO containing water at a concentration of 1 mg/ml and 20 mg/ml, respectively. Further dilutions were performed in medium. Monoclonal antibodies specific for DC surface molecules were directly labelled and purchased from Becton Dickinson (Heidelberg, Germany). The unlabelled CCR7 (clone 2H4) antibody (Becton Dickinson) and the MART-1/Melan-A antibody (clone A103; Dako Cytomation, Hamburg, Germany) were detected with the additional use of secondary antibodies [Cy5-coupled F(ab')_2_-antibody (Dianova, Hamburg, Germany) and biotinylated F(ab')_2_-antibody (Becton Dickinson)] and streptavidin-PE (Dianova). FITC-dextran from Sigma-Aldrich (Deisenhofen, Germany) and CCL19 from R&D Systems (Wiesbaden, Germany) were used. IL-1β, IL-4, IL-6 and TNF-α were purchased from R&D Systems, IL-2 from Chiron Behring (Marburg, Germany), GM-CSF (Leukine^®^) from Berlex (Seattle, USA) and PGE_2 _from Sigma-Aldrich.

### Tumor cell lines and CTL

The melanoma cell lines Mel-93.04A12 (HLA-A2^+^, Melan-A^+^; gift from P. Schrier, Department of Immunohematology, Leiden University Hospital, Leiden, the Netherlands), Mel A375 (HLA-A2^+^, Melan-A^-^; CRL-1619; ATCC) and SK-Mel-29 (HLA-A2^+^; gift from T. Wölfel, Third Department of Medicine, Hematology and Oncology, Johannes Gutenberg University of Mainz, Mainz, Germany) were cultured in RPMI 1640 medium supplemented with 10% fetal calf serum, 2 mM L-glutamine, 1 mM sodium pyruvate and non-essential amino acids. AK-EBV-B cells (gift from T. Wölfel) were cultured in RPMI 1640, containing 10% fetal calf serum. The HLA-A2-restricted, MART-1/Melan-A_26-35 _specific CTL A42 (gift from M. C. Panelli, National Institutes of Health, Bethesda, MD) were cultured in RPMI 1640 supplemented with 10% human serum (Lonza, Walkersville, USA), 2 mM L-glutamin, 1 mM sodium pyruvate, 100 IU penicillin/streptomycin, 0.5 μg/ml mycoplasma removal agent (MP Biomedicals, Eschwege, Germany) and 125 IU/ml IL-2. 5 × 10^5 ^CTL were restimulated every two weeks using 1 × 10^5 ^SK-Mel 29 and 2 × 10^5 ^AK-EBV-B (both irradiated with 100 Gy) in 1.5 ml A42 CTL medium per well of a 24-well plate. On the day of restimulation, 500 IU/ml IL-2 were added to the culture. A42 CTL were used for coculture experiments 8 days after restimulation.

### Generation and culture of 3d DC and 7d DC

Monocytes were enriched from heparinized blood by Ficoll density gradient centrifugation and subsequent plate adherence or from a leukapheresis product via elutriation, as described previously [30]. For freezing of multiple aliquots, 2-4 × 10^7 ^monocytes per ampule were resuspended in VLE (very low endotoxin) RPMI supplemented with 5% human serum albumin (20% Octalbin^®^, Octapharma, Langenfeld, Germany) and mixed 1:1 with freezing medium, containing VLE RPMI, 10% human serum albumin and 20% DMSO. After thawing, 15 × 10^6 ^monocytes were plated in a Nunclon™flask (80 cm^2^; Nunc, Wiesbaden, Germany) in VLE RPMI medium supplemented with 1.5% human serum. For inducing the development of 2d iDC, 20 ng/ml IL-4 and 100 ng/ml GM-CSF were added to the medium immediately after plating the monocytes. On day two, 2d iDC could be harvested for study. For maturation, the 2d iDC were cultured with the four component cocktail, containing 10 ng/ml IL-1β, 15 ng/ml IL-6, 10 ng/ml TNF-α and 1000 ng/ml PGE_2 _in addition to 100 ng/ml GM-CSF and 20 ng/ml IL-4 [22]. After 24 h, the 3d mDC were harvested for study. To generate 7d DC, the culture medium was supplemented with 20 ng/ml IL-4 and 100 ng/ml GM-CSF on days 1 and 3 after plating the monocytes. On day 6, the maturation cocktail (as for 3d mDC) was added to the culture of 6d iDC and 7d mDC where harvested for study after 24 h. Prior to freezing, DC were resuspended in 20% human serum albumin and mixed with equal amounts of freezing medium, containing 20% human serum albumin, 20% DMSO and 10% glucose (Braun, Melsungen, Germany).

### Generation of MART-1/Melan-A *ivt*RNA

The mMESSAGEmMACHINE™Kit from Applied Biosystems (Darmstadt, Germany) was used for the production of MART-1/Melan-A *ivt*RNA. The linearized vector pcDNAI/Amp/Aa1 (gift from T. Wölfel), encoding the MART-1/Melan-A cDNA, served as a template for *in vitro *transcription. To increase the stability of the RNA, a poly-A tail was added to the *ivt*RNA with the aid of the Poly(A) Tailing Kit™(Applied Biosystems). The kits were used according to the manufacturers' instructions.

### Cell surface staining of DC

The expression of cell surface molecules on DC was detected using specific monoclonal antibodies [CD14 (clone M5E2), CD83 (clone HB15e), CD209 (clone DCN46), CD40 (clone 5C3), HLA-DR (clone G46-6), CCR7 (clone 2H4), CD86 (clone 2331), CD80 (clone L307.4) and CD274 (clone M1H1), all Becton Dickinson] and measured by flow cytometry. 5 × 10^4 ^DC were washed with ice-cold PBS supplemented with 1% FCS and incubated for 30 min with the appropriate antibody (1:25 dilution). If the first antibody was directly linked to a fluorochrome, the cells were washed once again, as described above, and resuspended in 200 μl PBS containing 1% FCS. If use of a secondary antibody was necessary, the cells were washed and incubated with the secondary antibody for an additional 20 min, washed again and resuspended as described above. The DC were analyzed using either FACS Calibur™or LSR-II™instruments (BD Biosciences, Heidelberg, Germany). Results were evaluated using the CellQuest™(BD Biosciences) or FloJo™(Tree Star, Inc., Ashland, OR) software.

### Intracellular staining of DC

For the detection of intracellular MART-1/Melan-A protein, 3 × 10^5 ^DC were fixed in PBS containing 1% paraformaldehyde (PFA) for 30 min on ice. After fixation, cells were washed with ice-cold PBS containing 1% FCS and resuspended in 500 μl 0.1% saponin in PBS (Sigma-Aldrich) to enable permeabilization of the cell membrane. The cells were centrifuged and the cell pellet subsequently resuspended in 0.25% saponin in PBS. The MART-1/Melan-A antibody was added to the cell suspension (dilution 1:20) and incubated for 1 h at room temperature. After incubation, the cells were washed twice in 0.1% saponin in PBS. Incubation with the secondary, Cy5-coupled antibody (dilution 1:100) was performed in 0.25% saponin in PBS for 30 min at room temperature. Before being resuspended in PBS with 1% FCS, the cells were washed in 0.1% saponin in PBS once again. The MART-1/Melan-A expression was analyzed by flow cytometry, as described for cell surface staining.

### Phagocytosis assay

The phagocytosis capacity of DC was tested via uptake of FITC-dextran. 2 × 10^5 ^DC were resuspended in 400 μl VLE RPMI containing 1.5% human serum, supplemented with 10 μg/ml FITC-dextran for 1 h at 37°C and 5% CO_2_. As controls, the same concentrations of DC were incubated in medium without FITC-dextran for 1 h at 37°C or in medium supplemented with 10 μg/ml FITC-dextran for 1 h on ice. After incubation, the cells were washed 3-4 times with ice-cold PBS containing 1% human serum and 0.1% NaN_3_. The cells were resuspended in PBS containing 1% human serum and analyzed by flow cytometry.

### Peptide-loading of DC

3-4 × 10^6 ^DC were incubated with different concentrations of the long or short MART-1/Melan-A peptides in a six-well-plate in VLE RPMI with 1.5% human serum. The incubation duration for the long peptide was 24 h and for the short peptide 2 h or 24 h. After incubation the DC were washed to remove excess peptide.

### Electroporation of DC

Electroporation of DC was performed with the Gene Pulser Xcell™from Biorad (München, Germany) in 0.4 cm electroporation cuvettes (Biorad). Prior to electroporation, DC were washed twice in ice-cold OptiMEM I medium (Invitrogen, Karlsruhe, Germany). 2-3 × 10^6 ^DC were resuspended in 200 μl OptiMEM I, preincubated on ice for three min and mixed with the MART-1/Melan-A *ivt*RNA (or the long MART-1/Melan-A peptide) in the electroporation cuvette. DC were pulsed with either 250 V, 150 μF or 300 V, 300 μF (exponential protocol). DC electroporated with H_2_O were used as controls. Directly after pulsing, the cells were transferred into a six-well-plate, containing VLE RPMI with 1.5% human serum, and incubated at 37°C and 5% CO_2 _for 24 h.

### Migration assay

A standard migration assay [31] was performed to determine the migratory capacity of DC. 2 × 10^5 ^DC were resuspended in 100 μl migration medium (RPMI 1640 supplemented with 1% human serum, 500 U/ml GM-CSF and 250 U/ml IL-4) and incubated in the upper chamber of a 24-trans-well-plate (Costar/Corning, USA) for 2 h at 37°C and 5% CO_2_. To determine chemokine-directed migration, the lower chambers contained 600 μl migration medium supplemented with 100 ng/ml CCL19 (R&D Systems). For detection of spontaneous migration and cell chemokinesis, the migration medium in the lower chamber either contained no CCL19 or CCL19 was present in both the upper and lower chambers. After 2 h of incubation the cells from the upper and lower chambers were harvested and cell counts determined with the aid of the CellTiter-Glo^® ^Luminescent Cell Viability Assay (Promega).

### Induction of antigen-specific T lymphocytes

3d and 7d mDC were harvested and pulsed with 10 μg/ml MART-1/Melan-A_26-35 _peptide (ELAGIGILTV) for 120 min at 37°C, 5% CO_2 _in a humidified atmosphere. Cryopreserved autologous PBMC isolated from HLA-A2^+ ^donors were cocultured with autologous, peptide-pulsed mDC using 1 × 10^6 ^PBMC and 1 × 10^5 ^mDC in T cell medium (RPMI 1640, 12.5 mM HEPES, 4 mM L-glutamine, 100 U/ml penicillin and streptomycin, supplemented with 10% pooled human serum). After 7 days of coculture, recovered lymphocytes were restimulated using the same cryopreserved batch of peptide-pulsed DC for 24 h, at which time supernatants were collected for determination of IFN-γ content via a standard ELISA using the OptEIA™Human IFN-γ ELISA Kit from BD Biosciences (Heidelberg, Germany) according to the manufacturers' protocol.

### Restimulation of effector CTL

A42 CTL were stimulated with tumor cells or antigen-loaded DC at a ratio of 2 × 10^4 ^CTL and 4 × 10^4 ^tumor cells/DC per 96-well in 200 μl A42 CTL medium. The coculture was set up 24 h after peptide-loading or pulsing of the DC with *ivt*RNA, if not otherwise indicated. The stimulation period was 24 h. Coculture supernatants were stored at -80°C for later analyses. The IFN-γ release of the stimulated A42 CTL was measured in the supernatant media by ELISA, as above.

## Results

### Morphology and FITC-dextran uptake of 3d mDC and 7d mDC

**Figure 1:**
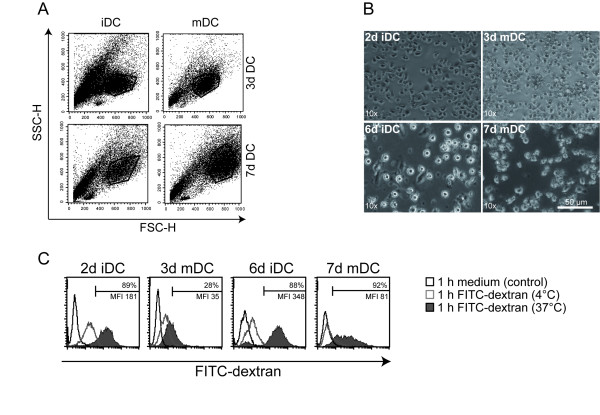
**Morphology and FITC-dextran uptake of 3d mDC and 7d mDC**. The size and the morphology of immature and mature 3d DC and 7d DC were analyzed by **(A) **flow cytometry and (**B) **light microscopy. **(C) **DC were incubated without or with 10 μg/ml FITC-dextran at 37°C or at 4°C for 1 hour. The cells were then washed three times in ice-cold PBS with 1% FCS. The uptake of FITC-dextran was analyzed by flow cytometry. Data are representative for three independent experiments. The left-most open histograms represent medium only controls, the open grey curves indicate mDC incubated with FITC-Dextran at 4°C and the filled histograms at 37°C.

Immature and mature DC were generated *in vitro *using either elutriated monocytes or monocytes obtained via plate adherence of freshly isolated PBMC of healthy donors. In all experiments, the 4C described by Jonuleit and colleagues was used for DC maturation [22]. Standard 7d mDC were induced within one week, whereas 3d mDC were generated within 72 hours. The different DC types were analyzed via flow cytometry and light microscopy and compared in terms of size and morphology (Fig. 1A and 1B). It was noticeable that 3d mDC were much smaller and showed a lower granularity than 7d mDC. 3d mDC were similar in size to 2d iDC, whereas 7d mDC were clearly larger than 6d iDC (Fig. 1A and 1B). Furthermore, 3d mDC displayed a higher yield and viability compared to 7d mDC (Table 1). All four DC types displayed capacity for macropinocytosis, following incubation with 10 μg/ml FITC-dextran for 1 h at 37°C. As controls, DC were incubated without FITC-dextran under the same conditions or with FITC-dextran for 1 h at 4°C. Subsequently, FITC-dextran expression was analyzed by flow cytometry (Fig. 1C). As expected, 2d iDC and 6d iDC showed greater FITC-dextran uptake and 6d iDC achieved a higher mean fluorescence intensity compared with 2d iDC, although comparable percentages of positive cells were seen. A somewhat lower FITC-dextran uptake was usually detected in 3d mDC compared with 7d mDC. Nevertheless, immature and mature DC from both protocols displayed capacity to take up particles (e.g. antigens) from their surroundings.

**Table 1:** Yield, purity and viability of immature and mature DC

	**2d iDC**	**6d iDC**	**3d mDC**	**7d mDC**
	
**Donor 1**				
Yield*	n.d.^+^	n.d.^+^	8%	4%
Purity ^#^	n.d.^+^	n.d.^+^	39%	34%
Viability ^$^	n.d.^+^	n.d.^+^	94%	78%
				
**Donor 3^&^**				
Yield*	12%^§^	6%^§^	18%	9%
Purity^#^	57%	58%	60%	70%
Viability^$^	93%	84%	95%	86%
				
**Donor 4^&^**				
Yield*	n.d.^+^	n.d.^+^	4%	3%
Purity^#^	32%	30%	42%	60%
Viability^$^	85%	76%	86%	81%

* Yield: from PBMC with the starting population set at 100%.

^+ ^n.d.: not determined

^# ^Purity: SSC/FSC

^$ ^Viability: propidium iodide (PI) stain

^&^Donors 3 and 4 are identical with donors 3 and 4 in Table 2 and Table 3.

^§ ^These values are lower compared to 3d and 7d mDC due to cell loss from strong adherence of iDC.

### Phenotype of immature and mature DC

**Figure 2:**
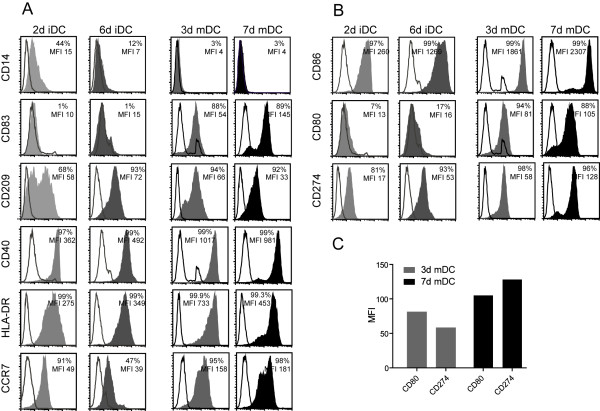
**Phenotype of immature and mature DC**. The expression of cell surface molecules on 2d iDC, 6d iDC, 3d mDC and 7d mDC was detected with specific antibodies and analyzed by flow cytometry. The open histograms correspond to the isotype controls, whereas the grey and black histograms display the specific binding of FITC- or PE-coupled antibodies. **(A) **Expression of CD14, CD83, CD209, CD40, HLA-DR and CCR7. **(B) **Expression of the B7-family-members CD86, CD80 and CD274. **(C) **Comparison of the expression of the costimulatory molecule CD80 and the inhibitory molecule CD274 on 3d mDC and 7d mDC. Data are representative for three independent experiments.

After 2, 3, 6 and 7 days, respectively, fast DC and standard DC were stained with monoclonal antibodies specific for cell surface molecules typically expressed on iDC and mDC and subsequently analyzed via flow cytometry. 2d iDC and 6d iDC displayed no CD83 and only very low expression of CD80, which is typical for iDC. Differences between 2d iDC and 6d iDC were seen in the expression pattern of CD14, CD209 (DC-SIGN), CD86 and CCR7 in various donors (n = 3). 3d and 7d mDC showed the expected mature phenotypes, with high expression of CD83 and no expression of CD14. Both also expressed high levels of costimulatory molecules, like CD80, CD86 and CD40, as well as other cell surface molecules that are important for the function of mDC, including CD209 (DC-SIGN), HLA-DR and CCR7 (Fig. 2A and 1B). Despite the shorter culture time, 3d mDC often expressed higher levels of CD209, CD40 and HLA-DR as compared to 7d mDC. Whereas higher expression of these molecules on 3d mDC varied among different donors, HLA-DR was consistently seen to be better expressed on 3d mDC in different donors (n = 3). In contrast, expression of the inhibitory molecule CD274 (B7-H1) was consistently lower on 3d than on 7d mDC. This difference was even more striking when the expression of the positive costimulatory molecule CD80 and the inhibitory molecule CD274 was directly compared. Thus, 3d mDC displayed a stronger positive costimulatory phenotype, with higher expression of CD80 compared to CD274, whereas 7d mDC showed a lower level of CD80 compared to CD274 (Fig. 2C). Despite variability in levels of expression among different donors, these data may suggest that 3d mDC might have a slight advantage in the expression pattern of costimulatory molecules and thereby may display a higher stimulatory capacity for T cells compared to 7d mDC.

### Migratory capacity of 3d mDC and 7d mDC

**Figure 3:**
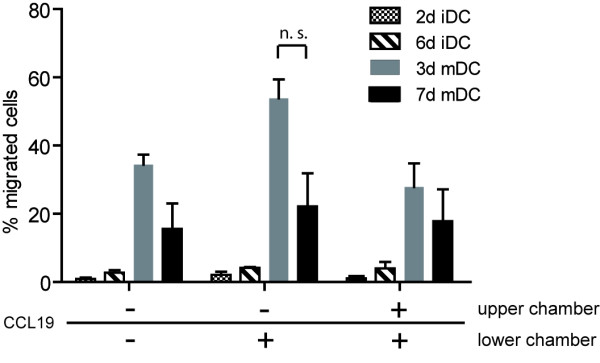
**Migratory capacity of immature and mature DC**. 2d iDC, 6d iDC, 3d mDC and 7d mDC were compared for their migratory capacity towards migration medium containing (+) or lacking (-) CCL19 in a trans-well migration assay. To measure directed migration, the medium in the lower chamber of the trans-well plate was supplied with 100 ng/ml CCL19, spontaneous migratory capacity was detected using medium that did not contain CCL19 in the lower chamber and random cell chemokinesis was determined by adding CCL19 to both the upper and the lower chambers. 2 × 10^5 ^DC were added in the upper chamber and incubated at 37°C and 5% CO_2 _for 2 h. Afterwards, DC numbers in the lower chambers were determined. Shown are three independent donors as mean values with standard errors of the mean (SEM). Statistical analyses were performed using the Mann Whitney test (n.s.: not significant).

One of the key features of DC, besides their ability to take up antigens in the periphery, is to migrate to the lymph nodes in order to present antigenic peptides to T cells. Both 3d and 7d mDC showed a high expression of CCR7 (Fig. 2A), which is an important receptor for homing of DC to lymph nodes. To test migratory capacity, iDC and mDC were examined using a standard migration assay. DC were incubated in the upper chamber of a trans-well-plate at 37°C for 2 h. The lower chambers contained migration medium, with or without the chemokine CCL19. As an additional control for cell chemokinesis, CCL19 was placed in both the upper and lower chambers. Since CCL19 is a specific ligand for the CCR7 receptor, migration of DC towards medium containing CCL19 reveals a directed migratory capacity, whereas migration towards medium alone or in the presence of chemokine in both chambers corresponds to spontaneous, undirected migratory capacity and a more random movement of the DC (Fig. 3). Neither 2d iDC, nor 6d iDC showed an ability to migrate, even though some expression of CCR7 was detected on these immature DC (Fig. 2A). In contrast, 3d mDC showed a higher directed migration compared with spontaneous migration. Strikingly, 7d mDC showed reduced directed migration compared to 3d mDC in all five donors tested, although the CCR7 expression on 3d mDC and 7d mDC was nearly the same (Fig. 2A). However, the differences in the directed migratory capacity between 3d and 7d mDC were not statistically significant.

### MART-1/Melan-A peptide recognition on DC by A42 CTL

**Figure 4:**
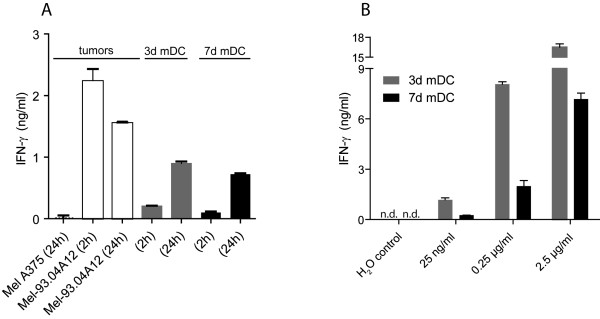
**Recognition of MART-1/Melan-A peptide on 3d mDC and 7d mDC by MART-1/Melan-A-specific CTL**. **(A) **3d mDC and 7d mDC were exogenously loaded with 10 μg/ml short MART-1/Melan-A_26-35 _peptide for 2 h or 24 h at 37°C and 5% CO_2_. After washing, the peptide-loaded DC were cocultured with MART-1/Melan-A-specific A42 CTL for 24 h at 37°C and 5% CO_2_. MART-1/Melan-A-positive tumor cells (Mel-93.04A12) and MART-1/Melan-A-negative tumor cells (Mel A375) served as controls and were cocultured with A42 CTL at the same time points as the DC (2 h and 24 h). The IFN-γ release of A42 CTL was measured by IFN-γ-ELISA. The columns show mean values of triplicates with standard deviations. Data are representative for two experiments. (**B) **3d mDC and 7d mDC were incubated with different amounts of long MART-1/Melan-A peptide for 24 h at 37°C and 5% CO_2_. The DC were cocultured with A42 CTL for additional 24 h. The IFN-γ release of A42 CTL was measured by IFN-γ-ELISA. The columns show mean values of duplicates with standard deviations. The data for 2.5 μg/ml are representative for two independent experiments (n.d.: not detected).

Next, 3d and 7d mDC were tested for their stimulatory capacity of CD8^+ ^effector T cells. Fast and standard mDC prepared from HLA-A2^+ ^donors were loaded exogenously with short MART-1/Melan-A_26-35 _peptide (ELAGIGILTV) for 2 h or 24 h. Because this peptide is only 10 amino acids long it can bind directly to HLA-A2 molecules. The peptide-loaded DC were cocultured for another 24 h with the MART-1/Melan-A-specific effector CTL A42 which recognize the MART-1/Melan-A_26-35 _peptide presented by HLA-A2 molecules. Activation of CTL A42 was measured by IFN-γ release. The MART-1/Melan-A-negative melanoma cell line Mel A375 and the MART-1/Melan-A-positive melanoma cell line Mel-93.04A12 were used as controls (Fig. 4A). A42 CTL showed IFN-γ release after stimulation with either 3d or 7d mDC. The amount of IFN-γ was higher in cocultures using DC that had an increased duration of peptide loading, indicating that 24 h of peptide loading provided DC with higher amounts of HLA-A2-peptide ligand, resulting in better stimulatory capacity. The 3d mDC were comparable to 7d mDC in their capacity to restimulate effector CTL after exogenous peptide loading for 24 h.

### Uptake of long MART-1/Melan-A peptide by 3d mDC and 7d mDC

The ability of 3d and 7d mDC to take up, process and present antigen was also tested. For this purpose, a long MART-1/Melan-A peptide, consisting of 23 amino acids, was used. This peptide is too long to be exogenously loaded directly onto HLA-A2 molecules. Therefore, it has to be processed by the DC, including cleavage by the proteasome and transport to the endoplasmic reticulum for binding on MHC and export to the cell surface, where it can be recognized by CTL. 3d and 7d mDC were incubated with different amounts of long peptide for 24 h, washed and cocultured with A42 CTL for an additional 24 h. IFN-γ release by the CTL was measured via ELISA (Fig. 4B). Again, both mDC types showed capacity to stimulate CTL after incubation with the long peptide, revealing that adequate uptake of peptide occurred and both DC types were able to intracellularly process and present the correct epitope.

### Electroporation of 3d mDC and 7d mDC with peptide or *ivt*RNA

**Figure 5:**
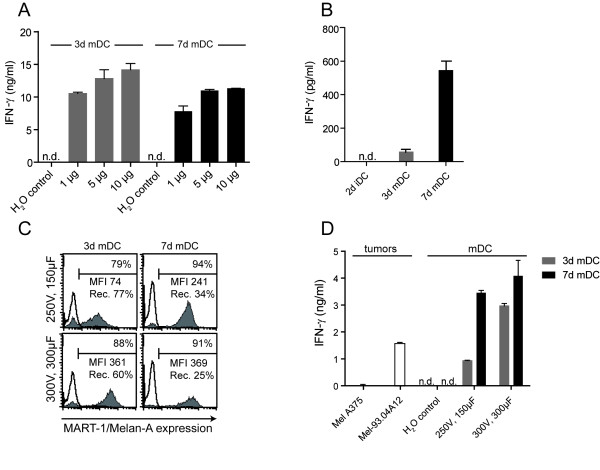
**Electroporation of 3d mDC and 7d mDC with long MART-1/Melan-A peptide and MART-1/Melan-A-encoding *ivt*RNA**. **(A) **3d mDC and 7d mDC were electroporated (250 V, 150 μF) with 1 μg, 5 μg and 10 μg long MART-1/Melan-A peptide. After 24 h incubation at 37°C and 5% CO_2_, the DC were cocultured with A42 CTL for 24 h. **(B) **2d iDC, 3d mDC and 7d mDC were electroporated (250 V, 150 μF) with 24 μg MART-1/Melan-A *ivt*RNA, incubated at 37°C for 24 h and cocultured with A42 CTL for 24 h (n = 3). **(C) **3d mDC and 7d mDC were electroporated with 12 μg MART-1/Melan-A *ivt*RNA at different electroporation conditions (250 V, 150 μF and 300 V, 300 μF), respectively. 3 h after electroporation, mDC were stained intracellularly with a MART-1/Melan-A-specific antibody and analyzed by flow cytometry (n = 2). **(D) **24 h after electroporation with MART-1/Melan-A *ivt*RNA, DC were cocultured with A42 CTL for 24 h (n = 2). The IFN-γ release of the A42 CTL was measured by IFN-γ-ELISA. The bars in A, B and D show mean values of triplicates with standard deviations (Rec.: recovery; n.d.: not detected).

To bypass the lower spontaneous uptake of antigen by mDC, it is possible to use electroporation to introduce either peptide or *ivt*RNA into DC. We compared 3d and 7d mDC that were electroporated according to optimal parameters that were previously established for 7d mDC [32]. After introduction of 1 μg, 5 μg or 10 μg of long peptide, the mDC were incubated at 37°C for 24 h and then cocultured with A42 CTL. Once again, 3d mDC showed capacity comparable to 7d mDC for stimulation of IFN-γ release by the CTL (Fig. 5A). Use of *ivt*RNA is an attractive source of antigen that can be easily and cheaply generated from any antigen-encoding cDNA. To analyze this as a source of antigen, immature and mature DC were electroporated with 24 μg of *ivt*RNA encoding MART-1/Melan-A, using the same electroporation conditions as applied with the long peptide. After 24 h of incubation at 37°C, the electroporated DC were cocultured with A42 CTL for another 24 h. Whereas 2d iDC were unable to stimulate A42 CTL, 3d mDC showed a weak but detectable stimulatory capacity. In contrast, A42 CTL responded very well to stimulation with *ivt*RNA-transfected 7d mDC (Fig. 5B).

Since the electroporation conditions used in this experiment were originally established for 7d mDC, it was possible that they might be suboptimal for 3d mDC. It was seen, for example, that the stimulatory capacity of 3d mDC could be improved by using higher amounts of *ivt*RNA with these electroporation parameters (data not shown). This, however, was a poor solution for clinical application of mDC since it would increase costs for production of *ivt*RNA. Therefore, alternate electroporation conditions for 3d mDC were explored in order to improve the efficiency of *ivt*RNA transfer. After testing several variations of electroporation, modified parameters of 300 V and 300 μF (exponential protocol) were found that facilitated optimal eGFP expression in 3d mDC after transfer of *ivt*RNA (data not shown).

Based on these observations, protein expression and stimulatory capacity were again compared in 3d and 7d mDC that were loaded with MART-1/Melan-A *ivt*RNA, applying both the old and modified parameters. Hereby, 3d and 7d mDC were electroporated with 12 μg *ivt*RNA, incubated for 24 h and then cocultured with A42 CTL for an additional 24 h. Three hours after electroporation, the MART-1/Melan-A protein expression was assessed in 3d and 7d mDC via intracellular staining using a MART-1/Melan-A-specific antibody and flow cytometry (Fig. 5C). With the modified parameters (300 V, 300 μF), 3d mDC showed a higher percentage of positive cells (88% vs. 79%) and a nearly five-fold increase in MFI (361 vs. 74) compared with 3d mDC electroporated according to the older conditions. In contrast, the percentage of MART-1/Melan-A positive cells remained similar with only a slight increase in MFI (1.5-fold) in 7d mDC. Under both conditions, 7d mDC displayed a poor recovery rate 24 h after electroporation, using either the old or modified parameters (34% and 25%, respectively) compared to 3d mDC (77% and 60%, respectively). Furthermore, 3d mDC showed a higher viability after electroporation compared to 7d mDC (Table 2). The improved MART-1/Melan-A expression in 3d mDC correlated with a substantial increase in stimulatory capacity (Fig. 5D). This was detected as a three-fold higher IFN-γ release from A42 CTL. 7d mDC also showed a somewhat higher stimulatory capacity, corresponding to their higher level of protein expression.

**Table 2:** Viability after electroporation, cryopreservation and thawing

		**3d mDC**			**7d mDC**		
			
		**w/o EP**	**300 V 300 μF**	**250 V 150 μF**	**w/o EP**	**300 V 300 μF**	**250 V 150 μF**
	
**Donor 3**	**Before freezing**						
	Cell counts (× 10^6^)	1.4	0.5	0.8	0.7	0.5	0.4
	Viability*	94%	90%	91%	75%	52%	74%
							
	**After thawing**						
	Cell counts (× 10^6^)	0.5	0.2	0.2	0.2	0.1	0.2
	Viability*	86%	79%	78%	51%	26%	55%
							
**Donor 4**	**Before freezing**						
	Cell counts (× 10^6^)	1.1	1.4	1.3	0.7	0.7	0.6
	Viability*	89%	85%	88%	59%	58%	62%
							
	**After thawing**						
	Cell counts (× 10^6^)	1.0	0.9	0.8	0.6	0.4	0.5
	Viability*	90%	90%	89%	86%	79%	84%

* Viability: PI stain

### Recoveries of 3d mDC and 7d mDC after freezing and thawing

For use in clinical application, it is important that large lots of antigen-loaded mDC can be prepared and cryopreserved in multiple aliquots for individual applications over time. To determine cell recovery after freezing and thawing, 3d and 7d mDC were frozen, without or 3 h after electroporation. After several days of storage, the DC were thawed and cell recoveries were determined (Table 2). In the absence of electroporation, the recoveries of both 3d and 7d mDC after cryopreservation and thawing were equal (68% vs. 70%, respectively). In contrast, 3d mDC displayed a greater robustness after electroporation and cryopreservation, leading to substantially higher cell recoveries compared with 7d mDC (41% vs. 18%) and to higher cell viabilities (Table 3).

**Table 3:** Recoveries of 3d mDC and 7d mDC after cryopreservation and thawing

	3d mDC - EP*		7d mDC - EP*		3d mDC + EP*		7d mDC + EP*	
				
	**counts** **(× 10**^**6**^**)**	**%**	**counts** **(× 10**^**6**^**)**	**%**	**counts** **(× 10**^**6**^**)**	**%**	**counts** **(× 10**^**6**^**)**	**%**
	
**Donor 1**								
Before EP*					2.0	100	2.0	100
Before freezing	1.6	100	1.2	100	1.4	70	1.3	65
After thawing	0.9	59	0.9	73	0.9	43	0.5	26
								
**Donor 2**								
Before EP*					1.5	100	1.5	100
Before freezing	1.2	100	0.5	100	1.0	65	0.3	23
After thawing	1.0	83	0.5	98	0.7	47	0.3	17
								
**Donor 3**								
Before EP*					2.0	100	2.0	100
Before freezing	1.4	100	0.7	100	0.5	25	0.5	25
After thawing	0.5	37	0.2	23	0.2	11	0.1	3
								
**Donor 4**								
Before EP*					1.5	100	1.5	100
Before freezing	1.1	100	0.7	100	1.4	93	0.7	45
After thawing	1.0	91	0.6	86	0.9	61	0.4	27
								
**mean % after thawing **± **SD^+^**		68 ± 24		70 ± 33		41 ± 21		18 ± 11

* EP: electroporation

^+ ^SD: standard deviation

### Stimulation of naïve T cells

**Figure 6:**
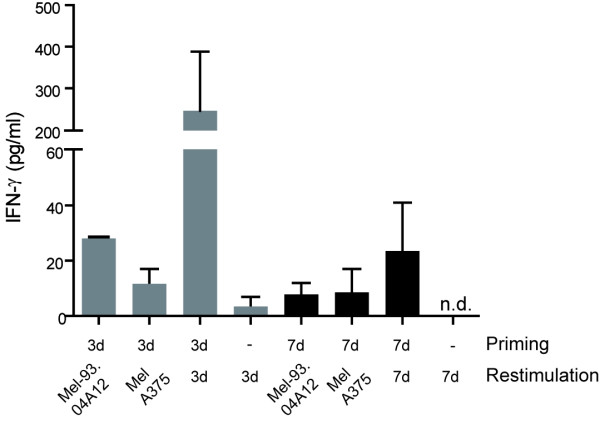
**Stimulation of naïve T cells with MART-1/Melan-A peptide-pulsed 3d and 7d mDC**. Autologous PBL were stimulated with MART-1/Melan-A peptide-pulsed 3d and 7d mDC for 7 days, followed by specific restimulation for 24 h using peptide-pulsed mDC and Melan-A-positive tumor cells. The IFN-γ release of the PBL was measured by IFN-γ-ELISA. Shown are two independent donors as mean values with SEM (n.d.: not detected).

Since it is essential for DC-based vaccines to enable *de novo *priming of new T cell responses, we also analyzed the capacities of MART-1/Melan-A peptide-loaded 3d and 7d mDC to stimulate naïve T cells in autologous cocultures. PBL were primed for seven days using either MART-1/Melan-A peptide-loaded 3d or 7d mDC. At this time the primed cells were recovered and restimulated with either melanoma tumor cell lines or with the same batches of peptide-pulsed 3d and 7d mDC that were cryopreserved and thawed before use as stimulating cells. The levels of IFN-γ secretion were detected by standard ELISA. When the primed T cells were restimulated with MART-1/Melan-A-expressing tumor cells they showed low levels of cytokine release which increased substantially upon restimulation with MART-1/Melan-A peptide-pulsed 3d mDC (Fig. 6). In contrast, the IFN-γ secretion of PBL stimulated with MART-1/Melan-A peptide-pulsed 7d mDC was much weaker. As described previously for DC that were matured with 4C cocktail, we did not detect any IL-12p70 secretion after stimulation of DC using CD40-ligand expressing cells from either 3d or 7d mDC (data not shown).

## Discussion

Since several different protocols for the generation of mDC using monocytes have been described to date, the aim of our study was to compare standard 7d mDC with 3d mDC in terms of phenotype, processing and presentation of antigen after transfection of either peptide or *ivt*RNA and stimulation of effector CTL. If 3d mDC display the same key characteristics as 7d mDC, they would be preferred for DC-vaccine development because of savings in time and costs.

In 2003, Dauer and colleagues published a protocol for the rapid generation of mDC *in vitro *[25,26]. These so called "fast DC" were induced from monocytes within 48 hours and showed typical phenotypic characteristics of mDC. We modified this procedure somewhat by adding the 4C maturation cocktail, designed by Jonuleit and colleagues [22], to the cultures of immature DC on the second day, thereby yielding mDC after three days of culture.

First, we observed that 3d mDC retained a smaller size and lower granularity compared to 7d mDC, as described for fast DC generated in 48 h [25,26,33]. This difference in morphology raised the issue whether 3d mDC would differ from 7d mDC in terms of antigen uptake, processing and presentation of antigen-derived peptides on the cell surface. Indeed, 3d mDC showed a lower capacity for spontaneous uptake of FITC-dextran from their surroundings, compared to 7d mDC. The phenotyping of 3d and 7d mDC revealed that both types of mDC expressed comparable levels of many important surface molecules. Nevertheless, some differences were observed in the levels of costimulatory molecules that play an important role in interactions with T cells. Thus, differences in the intensity of expression of the inhibitory molecule CD274 were detected, which may impact on the costimulatory capacities of 3d and 7d mDC for T cells. CD274 (B7-H1, PD-L1) on DC interacts with PD-1 on T cells and transmits an inhibitory signal [34,35]. Therefore, DC that express a preponderance of CD274 might inhibit rather than foster T cell activation. We observed that 7d mDC expressed higher levels of CD274 compared to the costimulatory molecule CD80. In contrast, 3d mDC showed a reciprocal lower expression of CD274 compared to CD80 (Fig. 2). These results indicate that 3d mDC may be more effective in activating T cells compared to 7d mDC, which would be advantageous for priming of naïve T cells. Nevertheless, when 3d and 7d mDC were loaded exogenously with short MART-1/Melan-A peptide, both DC types showed comparable capacities to stimulate A42 CTL (Fig. 4A). These findings indicated that the higher ratio of CD274 to CD80 in 7d mDC did not impair their capacity to stimulate primed effector cells (Fig. 2B and 1C). While DC loading with short peptide led to comparable stimulation of CTL, 3d and 7d mDC revealed different capacities to stimulate effector T cells after incubation with long peptide. IFN-γ release from T cells after stimulation with standard mDC loaded with long peptide was previously noted to be much higher than stimulation with standard mDC loaded with short peptide [36]. This may be due to maintenance of a persisting pool of long peptide within the DC that could be processed and presented for a longer period after uptake. In our studies 3d mDC showed equal or better stimulatory capacity for T cells after incubation with long peptide compared to 7d mDC, indicating that processing may have been more efficient in 3d mDC, since the spontaneous uptake of exogenous material was lower in 3d mDC, as evidenced by FITC-dextran uptake. After introduction of the long peptide by electroporation, comparable stimulatory capacities were found between 3d and 7d mDC. Since levels of IFN-γ release by T cells were in general lower after stimulation with DC that were electroporated with peptide compared with spontaneous peptide uptake, we speculate that electroporation might diminish somewhat the antigen processing capacity of the DC.

Next, we analyzed protein expression in 3d and 7d mDC after electroporation of *ivt*RNA, alongside their stimulatory capacity for CTL. As shown previously, antigen-loaded fast DC were clearly able to stimulate T cells [25,27]. However, we extended these observations by comparing the stimulatory capacities of 3d and 7d mDC side-by-side after electroporation of MART-1/Melan-A *ivt*RNA. Using previously established electroporation conditions, we noted that 3d mDC showed diminished stimulatory capacity compared to 7d mDC in repeated experiments with multiple donors. This was likely due to lower protein expression in 3d mDC after introduction of *ivt*RNA. The low protein expression in 3d mDC could be overcome by using greater amounts of *ivt*RNA (data not shown). However, based on the differences in morphology and size, we speculated that the more compact 3d mDC might be more robust and that more intense electroporation conditions might improve *ivt*RNA transfer, yielding better protein expression after transfer of lower amounts of *ivt*RNA. Indeed, alteration of the electroporation parameters yielded an improved protein expression in 3d mDC, which subsequently showed a much better stimulatory capacity for CTL. Thus, once 3d and 7d mDC expressed comparable levels of protein, they showed comparable stimulatory capacities for CTL.

When testing the migratory capacities of immature and mature 3d or 7d DC, we observed that immature DC displayed neither spontaneous nor chemokine-directed migration. This was also found by Dauer and colleagues for immature DC prepared in 24 h from monocytes [37]. 3d mDC appeared to have somewhat better migratory capacity than 7d mDC in multiple donors, which might be related to a less terminally-differentiated status. Since effective migration of DC is essential for the priming of naïve T cells in the lymph nodes, this characteristic supports use of 3d mDC for vaccine development.

Because 3d mDC were smaller than 7d mDC and seemed more resistant to electroporation, we speculated that they were more robust cells. Indeed, 3d mDC loading of *ivt*RNA required a stronger electroporation pulse to achieve similar protein expression to 7d mDC. Likewise, when recoveries of 3d mDC and 7d mDC were examined after cryopreservation and thawing, 3d mDC showed higher cell recoveries when the mDC were electroporated prior to cryopreservation. Furthermore, 3d mDC displayed higher cell viabilities after electroporation and cryopreservation when compared to 7d mDC. Thus, 3d mDC yielded higher cell recoveries and thereby would be superior to 7d mDC for clinical application if a DC vaccine strategy entails electroporation with antigenic proteins or antigen-encoding *ivt*RNA, followed by cryopreservation of multiple aliquots for thawing and immediate application to patients.

Since it is important that mDC are able to stimulate naïve T cells in a vaccine setting, we analyzed the stimulatory potential of MART-1/Melan-A peptide-pulsed 3d and 7d mDC on autologous PBL. Under short-term priming conditions of only seven days, we failed to detect any tumor-specific killing of MART-1/Melan-A-expressing tumor cells nor did we detect enrichment of MART-1/Melan-A-multimer-positive T cells (data not shown). Nevertheless, PBL stimulated with peptide-pulsed 3d mDC showed a higher IFN-γ release when restimulated with peptide-loaded 3d mDC compared to PBL that were primed and restimulated with 7d mDC. It has been shown previously by others, that fast DC generated in 48 h had an equal or greater capacity to stimulate naïve T cells compared to 7d mDC [28,33]. The failure to detect cytotoxic activity and detectable numbers of MART-1/Melan-A-multimer-positive T cells in our experiments is likely related to the use of 4C cocktail for DC maturation and the absence of IL-2 or IL-7 in the culture medium. It has already been described that DC matured with 4C cocktail do not produce bioactive IL-12p70, which is important for optimal polarization of naïve T cells for tumor recognition [30,38]. Improved stimulatory capacity of naïve T cells is achieved if mDC are generated using a cocktail that enables their production of IL-12p70. Recently, we showed indeed that 3d mDC, matured with TLR-ligand containing maturation cocktails display a much higher stimulatory capacity for naïve T cells than 3d mDC matured with 4C cocktail [38].

## Conclusions

Here we show that 3d mDC displayed similar characteristics to 7d mDC concerning phenotype and capacity to stimulate CTL after exogenous pulsing with short peptide. We observed that 3d mDC also had good capacities to stimulate CTL after uptake and processing of long peptide and they displayed a strong chemokine-directed migration. The 3d mDC were more robust and thereby required altered conditions for introduction of RNA-encoding antigen via electroporation, however this characteristic likely accounts for higher cell recoveries after electroporation and cryopreservation compared to 7d mDC. Thus, 3d mDC offer a suitable alternative to 7d mDC for use in clinical trials, thereby saving time and costs for cell production.

## Abbreviations

CCR: chemokine receptor (C-C type); CD: cluster of differentiation; CTL: cytotoxic T lymphocyte(s); DC: dendritic cell(s); EGFP: enhanced green fluorescent protein; FACS: fluorescence activated cell sorting; FCS: fetal calf serum; GM-CSF: granulocyte macrophage-colony stimulating factor; IDC: immature dendritic cell(s); IFN: interferon; IL: interleukin; *IVT*(RNA): *in vitro *transcribed RNA; MDC: mature dendritic cell(s); MHC: major histocompatibility complex; PBS: phosphate buffered saline; PGE_2_: prostaglandin E_2_; poly (I:C): polyriboinosinic polyribocytidylic acid; TGF: transforming growth factor; TNF: tumor necrosis factor; VLE: very low endotoxin.

## Declaration of competing interests

The authors declare that they have no competing interests.

## Authors' contributions

MB designed and performed the experiments and drafted the manuscript. SS and SW contributed to the initial development of 3d DC and provided scientific and technical advice. BF provided scientific advice and helped drafting the manuscript. DJS provided scientific advice, discussions of data and revised the manuscript. CG provided scientific advice, discussions of data and helped in the design of experiments. All authors read and approved the final manuscript.
